# Changes in hepatic triglyceride content with the activation of ER stress and increased FGF21 secretion during pregnancy

**DOI:** 10.1186/s12986-021-00570-3

**Published:** 2021-04-13

**Authors:** Jiayu Lu, Ying Gong, Xinhong Wei, Zhenyu Yao, Rui Yang, Jinxing Xin, Ling Gao, Shanshan Shao

**Affiliations:** 1 Department of Endocrinology, Shandong Provincial Hospital Affiliated to Shandong First Medical University, 544, Jing 4 Rd., Jinan, 250021 Shandong China; 2 Department of Endocrinology, Shandong Provincial Hospital, Cheeloo College of Medicine, Shandong University, Jinan, 250021 Shandong China; 3 Shandong Provincial Key Laboratory of Endocrinology and Lipid Metabolism, Jinan, 250021 Shandong China; 4 Shandong Institute of Endocrine and Metabolic Disease, Jinan, 250021 Shandong China; 5 Shandong Medical Imaging Research Institute, Shandong University, Jinan, 250021 Shandong China; 6 Experimental Animal Center, Shandong Provincial Hospital Affiliated to Shandong First Medical University, Jinan, 250021 Shandong China; 7 Scientific Center, Shandong Provincial Hospital Affiliated to Shandong First Medical University, Jinan, 250021 Shandong China

**Keywords:** Endoplasmic reticulum stress, FGF21, Pregnancy, Lipid metabolism

## Abstract

**Background:**

To meet the needs of foetal growth and development, marked changes in lipid profiles occur during pregnancy. Abnormal lipid metabolism is often accompanied by adverse pregnancy outcomes, which seriously affect maternal and infant health. Further understanding of the mechanism of lipid metabolism during pregnancy would be helpful to reduce the incidence of adverse pregnancy outcomes.

**Methods:**

Pregnant mice were euthanized in the virgin (V) state, on day 5 of pregnancy (P5), on day 12 of pregnancy (P12), on day 19 of pregnancy (P19) and on lactation day 2 (L2). Body weight and energy expenditure were assessed to evaluate the general condition of the mice. Triglyceride (TG) levels, the cholesterol content in the liver, liver histopathology, serum lipid profiles, serum β-hydroxybutyrate levels, fibroblast growth factor-21 (FGF21) levels and the levels of relevant target genes were analysed.

**Results:**

During early pregnancy, anabolism was found to play a major role in liver lipid deposition. In contrast, advanced pregnancy is an overall catabolic condition associated with both increased energy expenditure and reduced lipogenesis. Moreover, the accumulation of hepatic TG did not appear until P12, after the onset of endoplasmic reticulum (ER) stress on P5. Then, catabolism was enhanced, and FGF21 secretion was increased in the livers of female mice in late pregnancy. We further found that the expression of sec23a, which as the coat protein complex II (COPII) vesicle coat proteins regulates the secretion of FGF21, in the liver was decreased on P19.

**Conclusion:**

With the activation of ER stress and increased FGF21 secretion during pregnancy, the hepatic TG content changes, suggesting that ER stress and FGF21 may play an important role in balancing lipid homeostasis and meeting maternal and infant energy requirements in late pregnancy.

## Background

Pregnancy (or gestation) is the development of one or more offspring, known as embryos or foetuses, within the maternal uterus. To meet the needs of foetal growth and development, marked changes in lipid profiles occur during pregnancy [1]. During early pregnancy, anabolism plays a major role in augmented lipid deposition in most maternal tissues, which is associated with both hyperphagia and increased lipogenesis. In contrast, advanced pregnancy is an overall catabolic state that involves the accelerated breakdown of fat depots, hyperlipidaemia and insulin resistance [2]. In rats, all depots of the maternal body showed an increase in fat content, and the enlarged subcutaneous fat depot was found to account for the largest proportion of total stored fat (36%) [3]. In 1994, Sohlstrom A et al. found that the liver fat content in rats on day 14 of gestation was increased by nearly 20% compared with that in virgin (V) controls [4]. More importantly, abnormal levels of lipids and their metabolites during pregnancy are related to adverse neonatal outcomes [5].

To meet the energy and resource demands of reproduction, a series of changes take place; these changes include an increase in the capacity of the gastrointestinal tract [6] and an obvious increase in the level of lipoprotein lipase, which controls fat uptake by adipose tissue [2]. The liver, the central organ for lipid metabolism, plays a vital role in regulating the production and clearance of lipids. Therefore, changes in liver lipid metabolism are also expected during pregnancy. A previous study reported that the lipid uptake and transport capacities of liver cells and triglyceride (TG) secretion levels are increased throughout reproduction [7]. Moreover, liver X receptor (LXR) targets contribute to lipogenesis during early pregnancy by increasing hepatic expression of the lipogenic enzymes fatty acid synthase (FASN) and stearoyl-CoA desaturase 1 (SCD1) [8]. However, the current understanding of liver lipid metabolism during pregnancy is inadequate. Importantly, the changes that occur in the levels of regulatory factors throughout the entire pregnancy period have not been reported.

Perturbations in endoplasmic reticulum (ER) homeostasis interfere with protein folding in the ER leading to ER stress, which in turn activates the unfolded protein response (UPR) [9, 10]. BIP was the first ER-resident protein involved in protein folding shown to bind incompletely assembled immunoglobulin (Ig) intermediates and to be bound by three principal transmembrane sensors. The UPR is activated via three pathways: the inositol-requiring enzyme (IRE)-1α, protein kinase R-like ER kinase (PERK), and activating transcription factor (ATF)-6α pathways [11, 12]. Much evidence suggests that ER stress is a common feature of fatty liver [13–16]. A study reported five years ago greater expression of PERK and X-box binding protein 1 (XBP1), which are related to ER stress, in the bovine liver two weeks before calving [17]. Moreover, the mRNA levels of XBP1, an ER stress-induced UPR marker, in the liver were found to suddenly increase at calving [18]. Therefore, the UPR may be an important regulator of lipid metabolism during pregnancy.

Fibroblast growth factor-21 (FGF21) was identified in 2000 and named based on its membership in the FGF family [19]. While conventional FGFs act through an autocrine or paracrine mechanism, FGF21 belongs to an FGF subfamily whose members have hormone‐like actions [20]. Serum FGF21, which is mostly secreted by the liver, can be delivered to the pancreas or adipose tissue and cause a series of effects such as weight loss and improved insulin sensitivity. A relationship between FGF21 and hepatic lipid metabolism has been described in mice. Knockdown of FGF21 was shown to contribute directly to liver steatosis through inhibition of β-oxidation [21]. Recently, it was shown that the level of the novel metabolic hormone FGF21 increased suddenly at the onset of lactation in dairy cows or during late pregnancy in mice [22, 23]. We speculated that the change in FGF21 secretion during pregnancy is related to liver lipid metabolism.

In this study, we verified the presence of pronounced ER stress in early pregnancy and a sharp increase in FGF21 secretion in late pregnancy and showed that hepatic FGF21 secretion is associated with the coat protein sec23a of coat protein complex II (COPII) vesicles. Moreover, we suspect that the activation of ER stress plays an important role in augmenting hepatic lipid deposition during early pregnancy and that an increase in hepatic FGF21 secretion is crucial in curbing the continuous increase in lipid content during late pregnancy.

## Materials and methods

### Animals

Female C57BL/6 mice (7–8 weeks old) were obtained from Beijing Vital River Laboratory Animal Technology Co., Ltd., and housed in a room with controlled temperature of 22–24 °C under a 12 h light/dark cycle. All mice were fed and allowed to acclimatize for one week. The mice were mated with male breeders or kept in the virgin state. Pregnancy was confirmed by identification of a vaginal plug (the day that the vaginal plug was found was assumed to be day 1 of pregnancy.). Subsets of mice were euthanized in the virgin state (V), on day 5 of pregnancy (P5, early pregnancy), on day 12 of pregnancy (P12), on day 19 of pregnancy (P19, late pregnancy) and on lactation day 2 (L2) [7, 8, 23, 24]. This animal study was reviewed and approved by the Animal Ethics Committee (LCYJ: No. 2018–017) of Shandong Provincial Hospital, China.

### Assessment of mouse body metabolism using metabolic chambers

Mice were acclimated to an animal monitoring chamber (PhenoMaster, TSE Systems, Germany) for 24 h and then placed in individual metabolic chambers on days 17, 18 and 19 of pregnancy. Afterwards, oxygen consumption (V_O2_), carbon dioxide production (V_CO2_), the respiratory exchange rate (RER) and heat production were simultaneously measured for 2 days.

### Sample collection and preservation

After all mice had been fasted for 6 h and anaesthetized with 1% pentobarbital sodium, blood was collected from the mice by eyeball extirpation. Then, the serum was isolated after centrifugation at 3000 rpm for 15 min. The livers were divided into several parts which were fixed for two days in a 4% paraformaldehyde solution or flash frozen in liquid nitrogen and then stored in liquid nitrogen or at − 80 °C for subsequent analyses.

### Biochemical analysis

Serum TG, total cholesterol (TC), low density lipoprotein cholesterol (LDL-C), high density lipoprotein cholesterol (HDL-C), nonesterified fatty acid (NEFA) and β-hydroxybutyrate (β-HB) levels were measured using a Beckman Chemistry Analyser AU5800 System (Beckman Coulter, Tokyo, Japan).The level of serum FGF21 was measured using commercial immunoassay kit (mouse FGF-21 ELISA kit, Abcam, ab212160). The absorbance at 450 nm was determined using an ELISA microtiter plate reader (BioTek Inc., Winooski, VT, USA).

### Hepatic lipid content assay

Lipid extraction was performed as previously described [25]. Briefly, liver homogenates were prepared with liver tissues of the same weight after heating at 70 °C for 10 min and then centrifuged at room temperature at 2000 rpm for 5 min. Hepatic TG, TC and free cholesterol (FC) contents were measured using commercial kits (Applygen Technologies Inc. Beijing, China), and the values were normalized to the weight of the liver tissue by gram.

### Histology

Liver tissues fixed in a 4% paraformaldehyde solution were used for haematoxylin and eosin (H&E) staining. Tissue blocks were routinely dehydrated in graded alcohol, cleared with xylene, embedded in paraffin and cut into 5 μm slices. Frozen sections were used for oil red O staining. The tissue blocks were cut into 10 μm slices, then stained with oil red O for 10 min, washed, and counterstained with haematoxylin for 2 min. All pathological images were observed using a system incorporated in a microscope (Axiovert 100 M Zeiss, Germany).

### RNA isolation and quantitative real-time PCR (Q-PCR)

Total RNA was isolated from the liver tissue using RNAiso Plus reagent (Takara, Japan) according to the manufacturer's instructions. RT reactions were carried out using a PrimeScript RT reagent kit (TaKaRa). Real-time PCR was performed using the Roche 480 detection system and SYBR Green mix (Bestar qPCR Mastermix, DBI, Germany) following the manufacturer's protocol. β-Actin was employed as an endogenous control. Relative gene expression levels were quantified using the 2-ΔΔCt method, and the results are expressed as the fold change relative to the control. The PCR primers used are listed in Table 1.

**Table 1:** Primers for real-time PCR detection in mice

Gene	GenBank ID	Primer sequence (5′ – 3′)
SCD1	20,249	Forward AAGATATTCACGACCCCACC Reverse CAGCCGTGCCTTGTAAGTTC
ACC1	107,476	Forward GCTTATTGATCAGTTATGTGGCC Reverse CTGCAGGTTCTCAATGCAAA
FASN	14,104	Forward GTCCTGGGAGGAATGTAAACAG Reverse CGGATCACCTTCTTGAGAGC
CD36	12,491	Forward AAGCAAAGTTGCCATAATTGAGTC Reverse GGAAAGGAGGCTGCGTCTG
FABP1	14,080	Forward TCAAGCTGGAAGGTGACAATAA Reverse GTCTCCATTGAGTTCAGTCACG
FATP2	26,458	Forward CGAGACGAGACGCTCACCTA Reverse ACGAATGTTGTAGTTGAGGCAC
FATP5	26,459	Forward TCTATGGCCTAAAGTTCAGGCG Reverse CTTGCCGCTCTAAAGCATCC
MTP	17,777	Forward TGGTGAAAGGGCTTATTCTGTT Reverse TTGCAGCTGAATATCCTGAGAA
ApoB	238,055	Forward AAACATGCAGAGCTACTTTGGAG Reverse TTTAGGATCACTTCCTGGTCAAA
PPARα	19,013	Forward AAGGGCTTCTTTCGGCGAAC Reverse TGACCTTGTTCATGTTGAAGTTCTTCA
CPT1α	12,894	Forward TTGGGCCGGTTGCTGAT Reverse GTCTCAGGGCTAGAGAACTTGGAA
Hadha	97,212	Forward TGCATTTGCCGCAGCTTTAC Reverse GTTGGCCCAGATTTCGTTCA
ACOX1	11,430	Forward TAACTTCCTCACTCGAAGCCA Reverse AGTTCCATGACCCATCTCTGTC
Acaa1b	235,674	Forward CAGGACGTGAAGCTAAAGCCT Reverse CTCCGAAGTTATCCCCATAGGAA
Ech1	51,798	Forward GCTACCGCGATGACAGTTTC Reverse GCTCAGAGATCGAAGGCTGATG
Ehhadh	74,147	Forward ATGGCTGAGTATCTGAGGCTG Reverse ACCGTATGGTCCAAACTAGCTT
MLycd	56,690	Forward GCACGTCCGGGAAATGAAC Reverse GCCTCACACTCGCTGATCTT
FGF21	56,636	Forward AGGATGGAACAGTGGTAGGCG Reverse GGCTTTGACACCCAGGATTTG
KLB	83,379	Forward TCCCCTGTGATTTCTCTTGG Reverse GAGCAATCTGTTGCCAGTGA
FGFR1	14,182	Forward CGCTCTACCTGGAGATCATT Reverse ATAAAGAGGACCATCCTGTG
FGFR2	14,183	Forward CACCAACTGCACCAATGAAC Reverse GGCTGGGTGAGATCCAAGTA
FGFR3	14,184	Forward CATCCGGCAGACATACACAC Reverse TTCACTTCCACGTGCTTCAG
FGFR4	14,186	Forward TAATGTTGGCAGTGTCAGGCCTCT Reverse TGGGAGCATAAGGCTGGAACAGAA
Yipf6	77,929	Forward TGTCAATTTCTCAGGACATTCCC Reverse CTCGCATAATGGTCCGACGA
Yipf5	67,180	Forward GGCTTTGATAACTTAAACAGCGG Reverse GTCACAGCCAGCATACTGCTT
Sar1a	20,224	Forward GATTGGACAATGCGGGCAAAA Reverse TCTGAAGTGGGATGGAGTGTT
Sec23a	20,334	Forward AGATGGGGTCCGGTTCAGTT Reverse GGTAGGTCGGGTCTCTCCTT
Sec24a	77,371	Forward CCACAAGTGTCATCGAGTCAA Reverse AGAACCACCGTAGTTCGACTG
Sec31a	69,162	Forward TGAACAGAGTGCCGAAGAAGA Reverse TGGTGACGTAATGGGAACAGG
Sec13	110,379	Forward TGAACACTGTGGACACCTCTC Reverse TTGACGGACCTATCCGAGGAG
β-actin	11,461	Forward ACCCCAGCCATGTACGTAGC Reverse GTGTGGGTGACCCCGTCTC

### RT2 PCR array

RNA extracted from the livers of mice in the V state and mice in late pregnancy were subjected to analysis with the Mouse Fatty Liver RT2 Profiler PCR Array (PAMM-157Z, Qiagen). The 96-well plate array was assessed using a Light Cycler 480 system (Roche) according to the manufacturer’s instructions to examine the expression of 84 genes related to fatty liver disease. Variations in gene expression between mice in late pregnancy and mice in the V state are expressed as fold changes. A detailed description of this PCR array can be found online (https://geneglobe.qiagen.com/cn/product-groups/rt2-profiler-pcr-arrays). The method for analysis of the liver RT2 Profiler PCR array was described in a previous study in our laboratory and another study [26, 27].

### Western blot analysis

Mouse liver tissues were homogenized in RIPA buffer with protease and phosphatase inhibitors (Bimake, Houston, USA). The protein concentration was determined using a BCA Protein Quantitative Assay Kit (Shenergy Biocolor Bioscience & Technology Co., Shanghai, China). Total protein was mixed with SDS loading buffer and subjected to SDS-PAGE on a 10% gel. Proteins were electrotransferred onto PVDF membranes (Millipore) and the blots were probed with the following primary antibodies overnight at 4 °C: anti-FASN (cst3180, Cell Signaling Technology, USA), anti-SREBP1C (ab3259, Abcam, USA), anti-SCD1 (ab19862, Abcam), anti-CPT1α (ab176320, Abcam), anti-MTP (sc-135994, Santa Cruz Biotechnology, USA), anti-CD36 (18836-i-ap, Proteintech, China), anti-FGF21 (ab171941, Abcam), anti-BIP (11587-1-ap, Proteintech), anti-p-IRE (ab48187, Abcam), anti-IRE (ab37073, Abcam), anti-eIF2α (cst9722, Cell Signaling Technology), anti-p-eIF2α (cst3597, Cell Signaling Technology), anti-ATF4 (10835-1-ap, Proteintech), anti-ATF6 (ab122897, Abcam), anti-p-PERK (sc-32577, Santa Cruz Biotechnology), anti-PERK (ab65142, Abcam) and anti-GAPDH (60,004–1, Proteintech). Appropriate secondary antibodies conjugated to horseradish peroxidase (Amersham) were diluted 1:5000 used. The bound primary antibodies were visualized using the Alpha Q detection system.

### Statistical analyses

All data are expressed as the means ± SEMs. Statistical significance was analysed using either two-tailed unpaired Student's t-tests (for two groups) or one-way ANOVA (for multiple groups). The data were analysed using SPSS 24.0 software. p < 0.05 was used to indicate statistical significance.

## Results

### Alterations in body energy expenditure, serum lipid levels and hepatic fat content during pregnancy

**Fig. 1:**
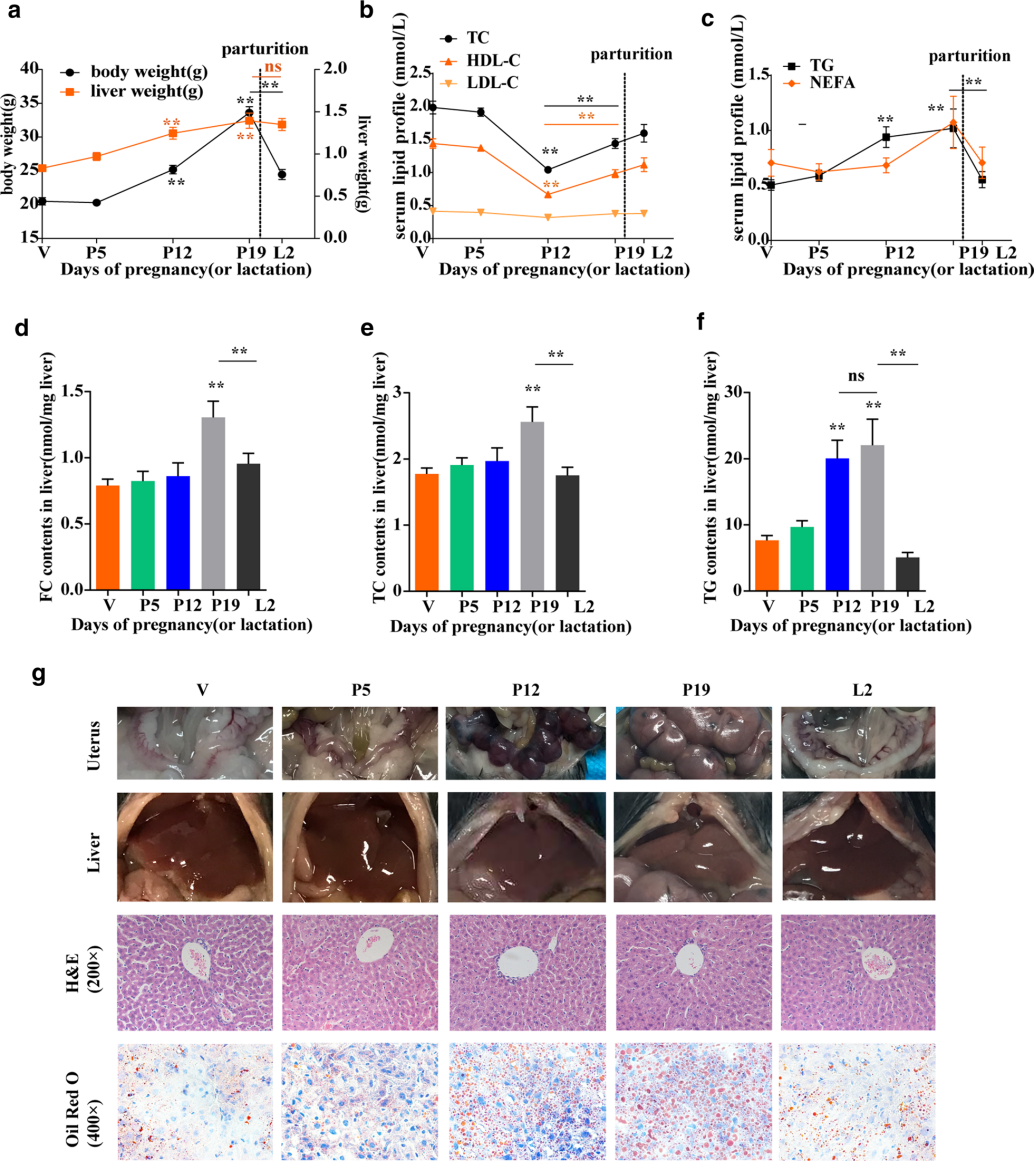
The effect of pregnancy on body weight, liver weight, the serum lipid profiles and lipid profiles of the liver. (**a**) Body weight and liver weight during pregnancy or lactation. (**b**) Serum TC, LDL-c and HDL-c levels. (**c**) Serum TG and NEFA levels. (**d**–**f**) TG content, TC content and FC content in the liver normalized by liver tissue weight in grams (n = 9–12 per group). (**g**) First row: Representative gross morphology of the uterus. Second row: Representative gross morphology of the liver. Third row: Representative images of H&E-stained liver tissue (magnification, 200 ×). Last row: Representative images of oil red O-stained liver tissue (magnification, 400 ×). Each bar represents the mean ± SEM of the indicated variable. Statistical analyses were performed with one-way ANOVA. *P < 0.05, **P < 0.01 versus the V or P19 group; ns, not significant

Gestation led to both body and liver weight gain especially during advanced pregnancy. Female mice showed a very rapid reduction in body weight after birth, while their liver weight was not significantly changed (Fig. 1a). To evaluate the effect of gestation on body energy metabolism, energy expenditure was measured using metabolic chambers. Compared with mice in the V group, mice in the P17-19 group, showed increased V_O2_, V_CO2_, and heat production, and a decreased RER, which indicated that β-oxidation was enhanced (Table 2). Moreover, the serum lipid levels of the mice differed during different gestation periods. TC and HDL-C levels reached a minimum in the P12 group and then gradually rose (Fig. 1b). To further examine the potential alterations in lipid levels in the liver, we used a hepatic lipid content assay and found that the hepatic cholesterol content rose in the P19 group but decreased in the L2 group (Fig. 1d, e), which differs from observations in humans; thus, these mice are not a good model of pregnancy in humans in which to study cholesterol metabolism. Then, we measured the TG level, which was obviously increased in the P12 and P19 groups compared to the V group (Fig. 1c); additionally, a significant increase in hepatic TG content was observed in the P12 and P19 groups (Fig. 1f). Consistently, more lipid droplets and ballooning degeneration were observed in the liver during late pregnancy, as shown by H&E and oil red O staining (Fig. 1g).

**Table 2:** Alterations in energy expenditure during pregnancy

	Non-pregnant	Late pregnancy
	V	P17-19
V_O2_ (ml/h)		
Light (12 h)	76.163 ± 0.525	104.413 ± 2.522^**^
Dark (12 h)	82.856 ± 0.422	108.279 ± 1.073^**^
Whole day (24 h)	79.714 ± 0.234	106.464 ± 1.028^**^
V_CO2_ (ml/h)		
Light (12 h)	69.098 ± 0.540	88.522 ± 1.547^**^
Dark (12 h)	78.779 ± 0.818	100.346 ± 1.135^**^
Whole day (24 h)	74.235 ± 0.583	94.796 ± 1.043^**^
Heat production (kcal/h)		
Light (12 h)	0.376 ± 0.003	0.509 ± 0.012^**^
Dark (12 h)	0.414 ± 0.002	0.538 ± 0.005^**^
Whole day (24 h)	0.396 ± 0.002	0.524 ± 0.005^**^
RER		
Light (12 h)	0.906 ± 0.003	0.849 ± 0.007^**^
Dark (12 h)	0.948 ± 0.010	0.924 ± 0.009
Whole day (24 h)	0.928 ± 0.005	0.889 ± 0.002^**^

Results are represented as mean ± SEM (n = 6–8) *p < 0.05, **p < 0.01, comparison of late pregnancy groups (P17-19) versus non-pregnant group (V). P value determined by unpaired t-test

### Throughout pregnancy, the expression of TG metabolism genes followed a distinct biphasic pattern

**Fig. 2:**
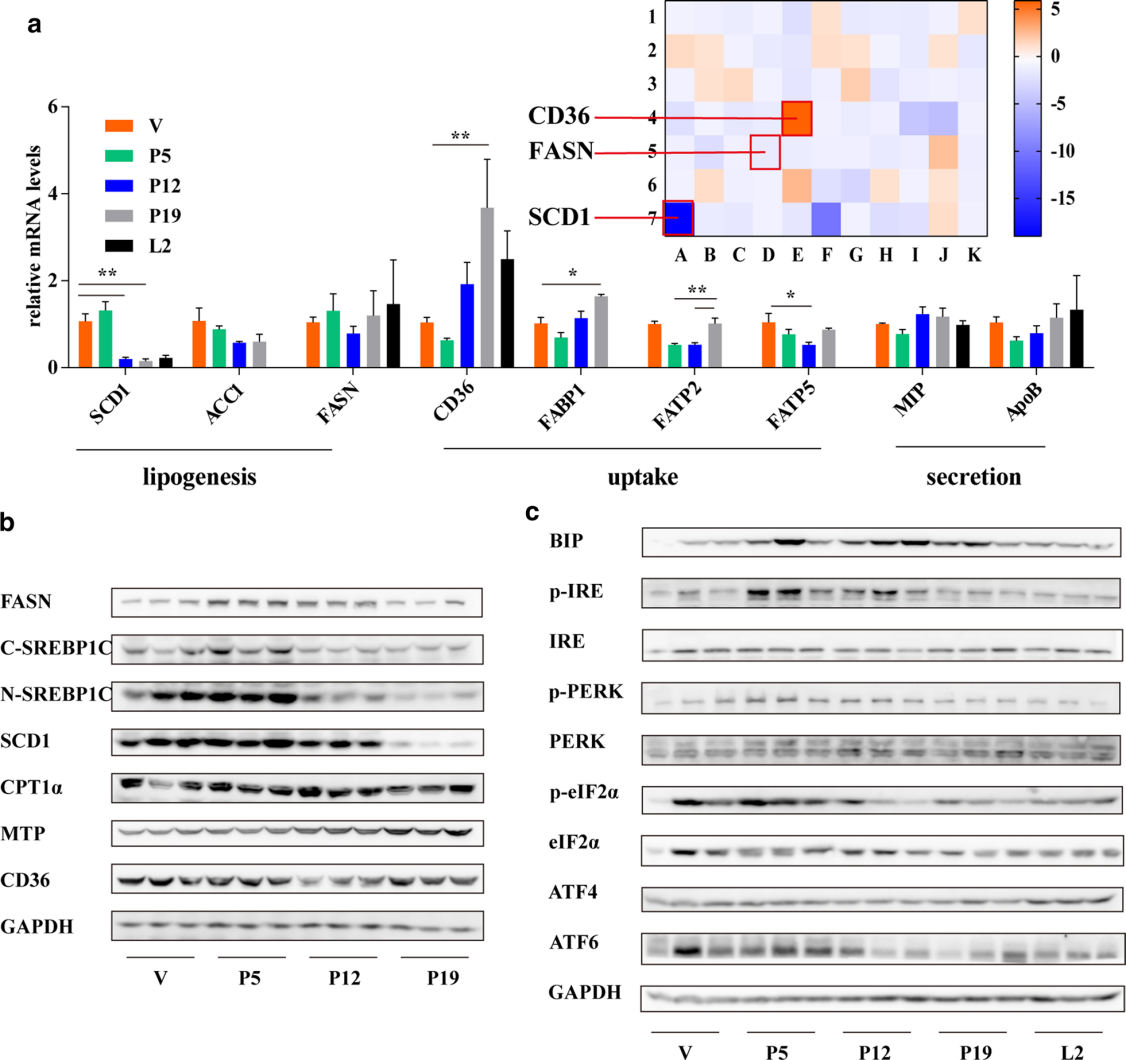
Changes in the hepatic expression of genes related to lipid metabolism and ER stress during pregnancy. (**a**) Transcript levels of liver TG metabolism-related genes during pregnancy or lactation (n = 3–7 per group). Microarray analysis showing the differential expression of genes in the liver between the late pregnancy groups (P17-19) and nonpregnant group (V) (n = 3 per group). (**b**) Protein levels of molecules related to TG metabolism in the liver. (**c**) Protein levels of molecules related to ER stress in the liver (n = 6 per group). Statistical analyses were performed with one-way ANOVA. *P < 0.05, **P < 0.01 versus the V or P19 group; ns, not significant

To further study the lipid metabolism mechanism during pregnancy, microarray analysis was performed to identify changes in murine hepatic gene expression between the late pregnancy groups and V group; we found that the expression of a set of lipid synthesis-related genes, including SCD1 and FASN, was downregulated in the late pregnancy group. The most striking change in lipid synthesis-related genes was the change in SCD1, the expression of which was markedly downregulated 19‐fold in the late pregnancy group compared with the V group (Fig. 2a). The qPCR results were consistent with these findings. In early pregnancy, consistent with the early-gestational increase in hepatic TG levels, the mRNA and protein levels of the lipogenic targets FASN and SREBP1C were markedly increased. In contrast, during late pregnancy, the mRNA and protein levels of SREBP1C, FASN and SCD1 were substantially diminished (Fig. 2a, b), which is consistent with the lack of a change in liver TG levels between P12 and P19 (Fig. 1f). In addition, the livers of the mice exhibited high lipid uptake and secretion, and liver transport ability was enhanced during pregnancy, as indicated by changes in expression of the related genes CD36, FABP1, FATP2 and FATP5 revealed by microarray analysis, qPCR and western blot analysis (Fig. 2a, b).

In addition, western blot analysis revealed that the gene expression of BIP and phosphorylation levels of IRE, PERK and eIF2a were increased in the P5 group compared to the V, P12 and P19 groups (Fig. 2c), indicating the possible activation of all three branches of ER stress during early pregnancy. Activation of any pathway of the ER stress response is an important cause of nonalcoholic steatohepatitis. We further found that the accumulation of hepatic TG did not occur until P12 (Fig. 1f), after the onset of overt ER stress, suggesting that ER stress may be involved in the accumulation of hepatic TG during pregnancy.

### Catabolism was enhanced with increased FGF21 secretion in the livers of female mice in late pregnancy

**Fig. 3:**
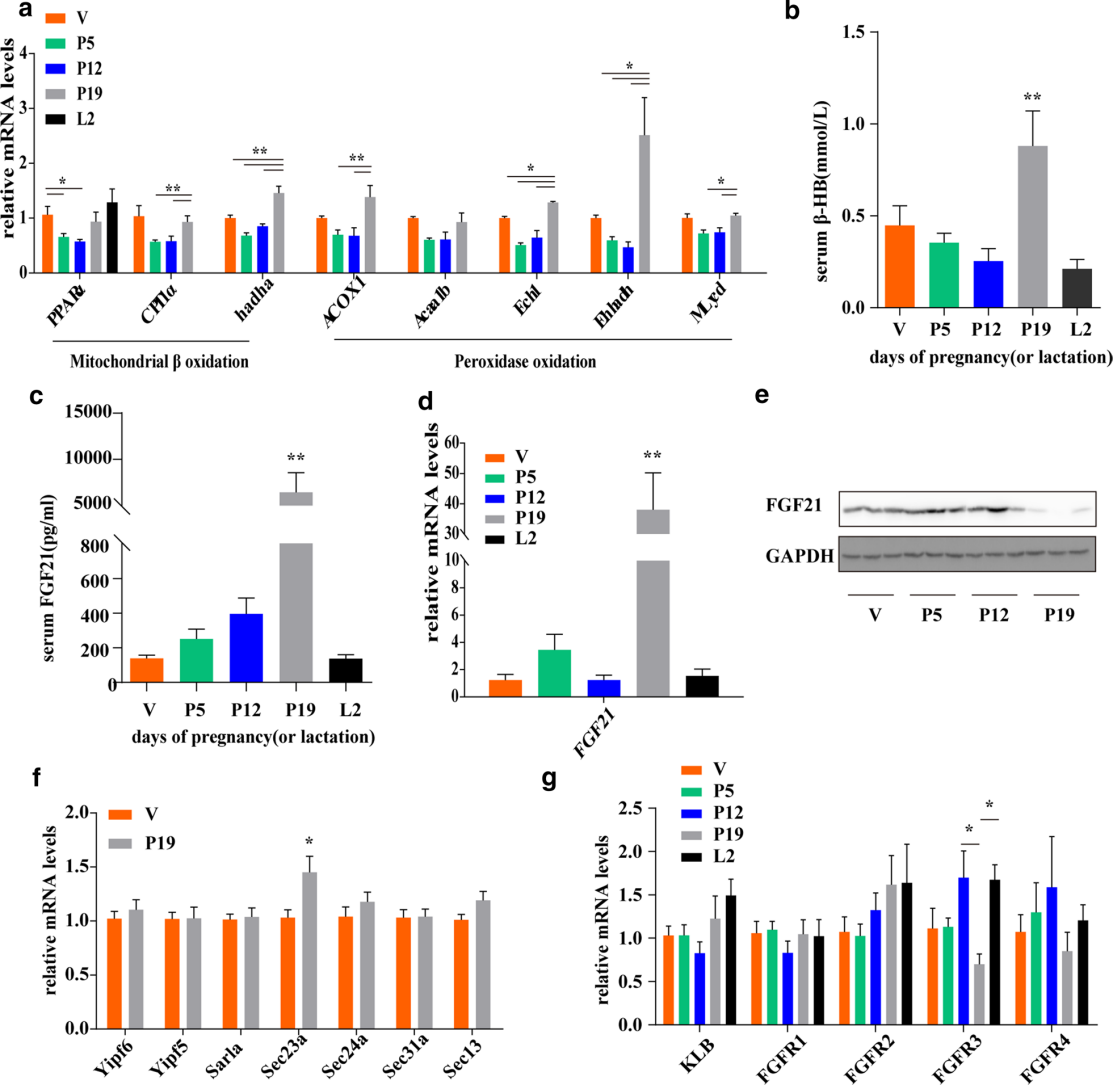
Alterations in β-HB and serum FGF21 levels; the expression of hepatic FGF21 and its receptors; and the expression of sec23a, a COPII vesicle coat protein during pregnancy. (**a**) Transcript levels of liver lipid oxidation related genes during pregnancy or lactation (n = 7 for the V, P5, P12 and P19 groups, n = 4 for the L2 group). (**b**) Circulating levels of β-HB during pregnancy (n = 9–12 for the V, P5, P12 and P19 groups, n = 5 for the L2 group). (**c**) Circulating levels of FGF21 during pregnancy (n = 9–12 per group). (**d**) Confirmation of changes in FGF21 mRNA expression during pregnancy by qPCR (n = 5–6 per group). (**e**) Protein levels of FGF21 (n = 6 per group). (**f**) Transcript levels of FGF21 secretion-related genes (n = 10–11 per group). (**g**) Transcript levels of KLB and FGFRs (n = 5–6 per group). Statistical analyses were performed with one-way ANOVA. *P < 0.05, **P < 0.01 versus the V or P19 group; ns, not significant

Levels of the β-oxidation related genes CPT1α,hadha,ACOX1,Ech1,Ehhadh and Mlycd, increased during late pregnancy (Fig. 3a), demonstrate that catabolism was dominant over anabolism. In addition, metabolic chamber analysis showed a decrease in RER in late pregnancy (Table 2). RER, the ratio of carbon dioxide produced to oxygen consumed, reflects the substrate consumed by the energy supply. With enhanced fatty acids oxidation, the RER decreases. Therefore, the metabolic chamber analysis results suggest that fatty acid oxidation in the liver was significantly enhanced.

In the livers of pregnant mice, fatty acid oxidation produces ketone bodies, the most important energy source for the foetus in the absence of a glucose supply. We found that the level of β-hydroxybutyrate, an important ketone body, was significantly increased in late pregnancy (Fig. 3b). In a ketotic state, the serum FGF21 level increased from slightly over 100 pg/ml in the V group to nearly 6000 pg/ml in the P19 group and had returned to the level in the V group by L2 (Fig. 3c). Furthermore, the mRNA expression of FGF21 significantly increased more than 30-fold at P19 and then returned to its former level at L2 (Fig. 3d). Surprisingly, hepatic FGF21 was almost undetectable in late pregnancy (Fig. 3e), which may have been related to increased FGF21 secretion. Therefore, we measured the gene expression of Yipf6, Yipf5, Sarla, Sec23a, Sec24a, Sec31a and Sec13 in secretory vesicles in the ER and Golgi [28]. The level of only sec23a, a small GTPase that initiates COPII coat assembly, was increased during late pregnancy (Fig. 3f). Moreover, the levels of the βKlotho (KLB) gene and the FGFRs genes FGFR1, FGFR2, FGFR3 and FGFR4 were measured. The expression levels of these genes, except FGFR3, were not affected by pregnancy (Fig. 3g).

## Discussion

Our main finding is that during early pregnancy anabolism plays a major role in augmenting hepatic lipid deposition, but advanced pregnancy is an overall catabolic state involving the accelerated breakdown of fat depots in the liver and hyperlipidaemia. Our study revealed that ER stress is a novel regulator during early pregnancy and that the increase in hepatic FGF21 secretion during late pregnancy is associated with Ses23a, a coat protein of COPII vesicles. Moreover, finding related to the novel hormone FGF21 will provide important insights into decreasing lipids and their metabolites during pregnancy to ensure maternal and child health.

Our results, established that gestation contributes to an increase in hepatic TG content in advanced pregnancy, and mice in the P12 and P19 groups exhibited larger lipid droplets than mice in the V group. We then assessed the metabolic rate, expressed as continuous V_O2_ consumption with a marked increase in V_CO2_ exhalation across a 24-h period, and found that body energy consumption was greatly increased during late pregnancy. In addition, increased heat production was confirmed. Notably, the RER was decreased, and more serum β-HB was produced, indicating an increase in fatty acid β-oxidation and a decrease in the amount of stored fat in late pregnancy compared to the V state. In summary, more lipids and fewer carbohydrates were oxidized in late pregnancy. TG accumulation in the liver likely suggests a balance between hepatic TG de novo lipogenesis, β-oxidation, and fatty acid uptake and secretion [29]. Notably, the lipid synthesis-related gene that showed the most striking decrease was SCD1, which regulates TG metabolism. The sharp drop in the expression of the lipid synthesis-related gene SCD1 and increase in expression of the β-oxidation-related genes CPT1α, hadha, ACOX1, Ech1, Ehhadh and Mlycd reflect the dominance of hepatic lipid catabolism in late pregnancy, providing lipids for foetal growth and development.

**Fig. 4:**
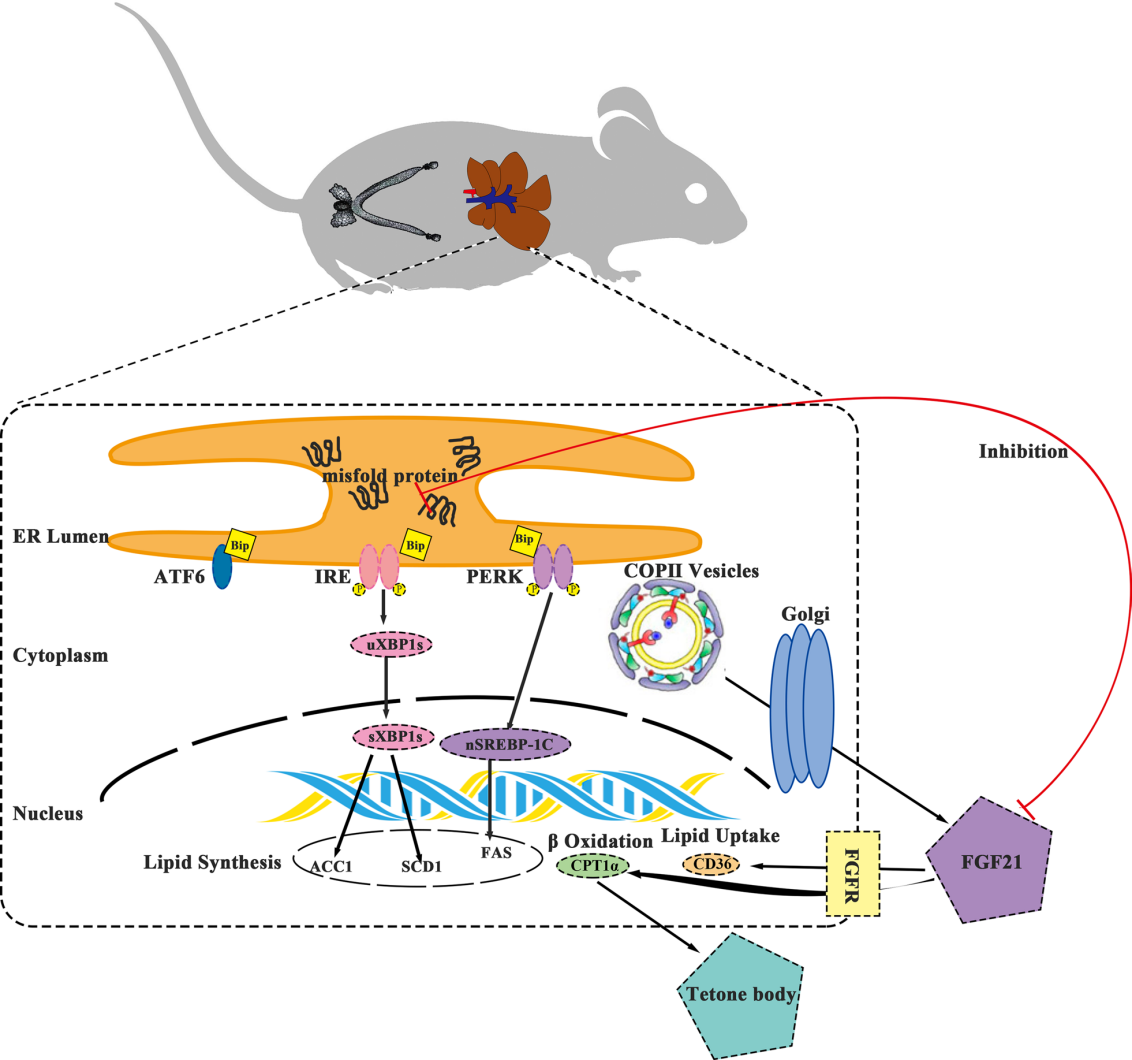
Schematic of the regulation and potential roles of ER stress and FGF21 in lipid metabolism during pregnancy. Activation of the PERK, IRE and ATF6 ER stress pathways plays an important role in augmenting hepatic TG synthesis and deposition during early pregnancy. A sharp increase in hepatic FGF21 secretion is crucial in curbing the continuous increase in hepatic TG content during late pregnancy, and hepatic FGF21 secretion is associated with the COPII vesicle coat proteins sec23a.

Previous studies have illustrated a simple relationship between ER stress and TG metabolism. The ER stress response was first discovered in nonalcoholic steatohepatitis, which is induced by genetics or diet [30]. The PERK-eIF2α pathway in ER stress has been reported to regulate lipogenesis and lipogenic differentiation, and deletion of PERK inhibited continuous expression of the lipogenic enzymes FASN and SCD1 [31]. Liver-specific knockout of IRE1α triggered severe hepatic steatosis after treatment with an ER stress inducer and suppressed the expression of key metabolic transcriptional regulators involved in TG biosynthesis [32]. ATF6α-knockout mice showed lower fatty acid β-oxidation, attenuated very-low-density lipoprotein (VLDL) formation and increased hepatic steatosis in response to an ER stress inducer [33]. Importantly, ER stress occurred earlier than hepatic TG accumulation in our pregnant mouse model. Therefore, we conclude that pronounced ER stress during early pregnancy is related to hepatic TG accumulation (Fig. 4).

Numerous hormones and nuclear receptors contribute to the regulation of hepatic lipid homeostasis [34–36]. Hormone levels change dramatically during pregnancy, and hormones are important factors related to changes in liver TG metabolism in this period. Oestrogen has been shown to contribute directly to alleviating fatty liver by disrupting insulin’s effects, promoting liver fat storage, reducing oxidative damage, and inhibiting TG synthesis [37, 38]. Prolactin inhibits hepatic steatosis by the CD36 pathway and reduces SCD1 gene expression [39, 40]. FGF21, a novel metabolic hormone is involved in coordinating diverse metabolic pathways to control glucose and lipid metabolism [19, 41]. In the mouse, FGF21 production is triggered by a flux of free fatty acids reaching the liver, arising from either the mobilization of lipid stores or consumption of a ketogenic diet [42, 43]. At present, the main regulatory factors of FGF21 that have been reported are PPARα and STAT5, which activate transcription of the FGF21 gene [44, 45]. On the other hand, the COPII coat is generated by a flexible vesicle formation system that drives ER export to the Golgi [46]. In this study, we found that the gene expression level of the Sec23a gene was increased, which has been confirmed to ensure the coordination of COPII vesicle trafficking and direction of ER–Golgi trafficking [47, 48]. Therefore, we hypothesize that the increase in FGF21 levels may induce a significant reduction in TG levels during late pregnancy (Fig. 4).

Interestingly, studies have shown that ER stress and FGF21 are strongly associated and inhibit one another [49]. We hypothesized that ER stress was dominant in early pregnancy while FGF21 synthesis was inhibited, causing the levels of lipid biosynthesis-related molecules to be increased in the livers of pregnant mice. However, FGF21 synthesis and secretion were then dominant; thus, fatty acid oxidation was enhanced in the livers of the pregnant mice, and liver lipid accumulation was no longer increased.

In addition to the abovementioned findings, insulin plays an important role in the regulation of glucose balance and metabolic homeostasis. Insulin stimulates glucose utilization; promotes protein synthesis and hepatic and muscle glycogenesis; and inhibits glucagon secretion, glycogenolysis and lipolysis [50]. Insulin-resistant individuals exhibit increased de novo lipogenesis and re-esterification, inducing fat accumulation in the liver [51]. According to reports in humans, total body insulin sensitivity is reduced by 45–70% in mid-to-late gestation [52, 53]. Studies in rodents also demonstrated that serum insulin level during late pregnancy gradually increased to approximately double that of the V state [54, 55]. Presumably, the results in our mice were similar, the serum insulin level increased in late pregnancy and may have contributed to increased TG accumulation in the liver in the P12 group. Notably, despite continuously increased insulin resistance, the hepatic TG content in the P19 group did not increase after P12. The sharp increase in FGF21 at P19 might be responsible for this.

The placenta is a major endocrine organ, and placental hormones have diverse and profound effects on maternal physiology and metabolism that should not be ignored. Experimental data from rodent models indicate that placental lactogen and prolactin can stimulate maternal beta cell proliferation during early gestation, increasing insulin secretion and aiding fat deposition [52]. Later in pregnancy, placental lactogen and growth hormone contribute to maternal insulin resistance and concomitantly increase de novo lipogenesis and re-esterification, inducing fat accumulation in the liver [53]. Thus, we cannot exclude the possibility that placental hormones and maternal hormones such as oestrogen, prolactin and insulin participate in hepatic TG metabolism in pregnancy.

As presented in this article, the main innovative founding of our study is the observation of changes in hepatic ER stress and FGF21 secretion during pregnancy. We inferred that ER stress was related to the increase in hepatic lipid content in early pregnancy, and that increased FGF21 secretion was related to the noncontinuous increase in the hepatic lipid content during late pregnancy. In addition, we are the first to use metabolic chambers to evaluate metabolic changes in mice during late pregnancy.

We did not use 4-phenylbutyric acid (4-PBA) to inhibit ER stress or knock down FGF21 in pregnant mice to explore the consequent changes in hepatic TG accumulation, which is a limitation of the present study. The causality of the reported relationship needs to be determined by further research.

## Conclusions

To the best of our knowledge, this is the first study to evaluate the activation of hepatic ER stress and FGF21 secretion during gestation and lactation. Our data reveal that the hepatic lipid content increases with the activation of ER stress in early pregnancy and that FGF21 secretion increases during late pregnancy, suggesting that ER stress and FGF21 may play important roles in balancing lipid homeostasis and meeting maternal and infant energy requirements in late pregnancy.

## Data Availability

All data generated or analyzed during this study are included in this published paper or are available from the corresponding author on reasonable request.

## Abbreviations

Acaa1b: Acetyl-CoA Acyltransferase 1b
ACC1: Acetyl-CoA carboxylase1
ACOX1: Acyl-CoA oxidase 1
ApoB: Apolipoprotein B
ATF: Activating transcription factor
BIP/GRP78: Immunoglobulin-binding protein
BMI: Body Mass Index
COPII: Coat protein complex II
CPT 1 α: Carnitine palmitoyl transferase 1
Ech1: Enoyl-CoA Hydratase 1
Ehhadh: Enoyl-CoA Hydratase and 3-Hydroxyacyl CoA Dehydrogenase
ELISA: Enzyme-linked immunosorbent assay
ER: Endoplasmic reticulum
FABP1: Fatty acid binding protein 1
FASN: Fatty acid synthase
FAT/CD36: Fatty acid translocase/Cluster of differentiation 36
FATP2: Fatty acid transport protein 2
FATP5: Fatty acid transport protein 5
FC: Free cholesterol
FGF21: Fibroblast growth factor 21
FGFR: Fibroblast growth factor receptor
GAPDH: Glyceraldehyde-3-phosphate dehydrogenase
Hadha: Hydroxyacyl-CoA Dehydrogenase Trifunctional Multienzyme Complex Subunit Alpha
HDL-C: High density lipoprotein cholesterol
H&E: Hematoxylin and eosin
ICP: Intrahepatic cholestasis of pregnancy
IRE: Inositol-requiring enzyme
KLB: βKlotho
L: Lactation
LDL-C: Low density lipoprotein cholesterol
LXR: Liver X receptor
Mlycd: Malonyl-CoA Decarboxylase
MTP: Mitochondrial trifunctional protein
NASH: Non-alcoholic steatohepatitis
NEFA: Non-esterified fatty acid
P: Pregnancy
PCR: Polymerase chain reaction
PERK: Protein kinase RNA-like ER kinase
PPARα: Peroxisome proliferators-activated receptor α
RER: Respiratory exchange rate
RNA: Ribose nucleic acid
SCD1: Stearyl coenzyme A desaturase 1
SREBP1C: Sterol regulatory element binding protein 1C
TC: Total cholesterol
TG: Triglyceride
UPR: Unfolded protein response
V: Virgin
**V**_**O**__**2**_: Oxygen consumption
V_CO__2_: Carbon dioxide production
VLDL: Very low-density lipoprotein
XBP1: X-box binding protein 1
β-BH: β-Hydroxybutyrate
4-PBA: 4-Phenylbutyric acid
